# Oral vaccination with inhibin DNA vaccine for promoting spermatogenesis in rats

**DOI:** 10.1590/1984-3143-AR2023-0079

**Published:** 2024-10-04

**Authors:** Jinzhu Meng, Jianhao Feng, Lilin Xiao, Nan Hu, Xianyong Lan, Shuilian Wang

**Affiliations:** 1 College of Veterinary Medicine, Hunan Agricultural University, Changsha, China; 2 Guizhou Provincial Key Laboratory for Biodiversity Conservation and Utilization in the Fanjing Mountain Region, Tongren University, Tongren, China; 3 College of Animal Science and Technology, Northwest Agriculture and Forestry University, Yangling, China

**Keywords:** inhibin, vaccine, immunization, spermatogenesis, rat

## Abstract

The objective of the present study was to evaluate the effects of a novel Inhibin (INH) DNA vaccine (C500/pVAX-asd-IS) on the immune response, reproductive hormone levels, and spermatogenesis of rats. Forty healthy male rats were divided into four groups, and respectively immunized (thrice, 14 d apart) with 1×10^8^, 1×10^9^, and 1×10^10^ CFU of the recombinant inhibin vaccine (group C500/pVAX-asd-IS-L, C500/pVAX-asd-IS-M, and C500/pVAX-asd-IS-H) or 1×10^10^ CFU C500. P/N values increased after vaccination and differed (*p* <0.05) at 7 d, and sharply increased at 14 d following the booster vaccination (*p* <0.01); The weight and volume of testes in C500/pVAX-asd-IS groups were increased (*p* < 0.05) at decapitation, respectively; Histological evaluation showed that the number of spermatogenic cells in the lumen was increased, and the cytoplasmic remnants of sperms were allergy increased significantly compared with the control group. Oral vaccination with INH DNA reduced (*P* < 0.05) serum concentrations of INH B, enhanced serum concentrations of testosterone (T) and FSH. Furthermore, mRNA expressions of *VIM* and *SMAD4* in the testes were increased in C500/pVAX-asd-IS-M and C500/pVAX-asd-IS-H groups (*p* < 0.05 or *p* < 0.01). The mRNA amount of *INHβ-B* in C500/pVAX-asd-IS-M group was greater than control group (*p* < 0.05).These results suggested that neutralization of endogenous INH through oral vaccination with INH DNA delivered by C500 strain successfully elicited a humoral immune response. INH gene immunization may have a positive effect on spermatogenesis and reproductive efficiency in male rats.

## Introduction

Inhibin (INH) is a glycoprotein hormone that is synthesized and secreted by sertoli cells of the testis and inhibits the secretion of follicle stimulating hormone (FSH) by the gonadotropic cells of the anterior pituitary in males (Petrozzi et al., 2019). INH contains two subunits (INH A and B), while INH B is the circulating isoform and only one detectable in adult males of most species (Li et al., 2018). The positive correlation of serum INH B concentrations, spermatogenesis, and testicular volume has been documented in many literatures (Bohring & Krause, 1999; Yalti et al., 2002). Serum INH-B concentrations were also positively correlated with total sperm counts (Jørgensen et al., 2010), and which appeared to be a reliable marker for human male fecundity (Ball et al., 2019).

DNA vaccine immunization is usually considered to be safe and effective which has been widely investigated (Dan et al., 2016a). Many studies have indicated that neutralization of endogenous INH through active immunization could improve reproductive efficiency, spermatogenesis and testicular development (Bame et al., 1999; Lovell et al., 2000). Immunization against INH enhances daily sperm production in urine of prepubertal rams (Al-Obaidi et al., 1987) and spermatid numbers in testes of bulls (Martin et al., 1991). Thus, these findings suggest that INH is a positive regulatory factor of spermatogenesis. However, there is a report suggesting that immunizing adult rams with INH vaccine increases serum FSH without altering sperm production (McKeown et al., 1997). The reason why INH immunization has no positive effect on sperm production in adult rams is unclear.

In the previous study, an attenuated Salmonella enterica serovar Choleraesuis C500 strain (*asd* and *crp* genes were deleted) was used as a delivery system for foreign antigens by using the *asd*^-^ balanced lethal chromosome–plasmid system (Zhao et al., 2009). Oral pharmacology with INH DNA vaccines expressing INH (1-32) fragment, which can enhance the number of larger follicles or litter sizes after immunization in mice and rats (Han et al., 2014; Wang et al., 2012). Dan et al., (2016a) successfully constructed a novel DNA vaccine p-TPA-SINH/TPA-SRFRP expressing INH-α (1–32), which exhibited better immunogenicity, and increased litter size of mice. However, there is little information regarding the INH DNA vaccine immunization on male animals. Consequently, we hypothesized that INH DNA vaccines exhibit promising effects on enhancing the reproductive performance of male rats. In this study, we aimed to evaluate the immune responses, reproductive hormone levels, testicular tissue structure, sperm count, sperm deformity rate, and spermatogenesis related gene expression through oral vaccination with INH DNA delivered by C500 strain in rats. Our findings will provide new insights for treating infertility caused by abnormal spermatogenesis.

## Methods

All animal experimental procedures were performed in accordance with relevant guidelines set by the Ethics Committee of Hunan Agricultural University, China (Approval number: 432019032).

### Recombinant plasmid

The attenuated *asd^-^* /*crp^-^ S. choleraesuis* C500 strain (C500) was stored in our laboratory. The kanamycin sequence in the expression vector pVAX1 (Invitrogen, Carlsbad, USA) was replaced by asd DNA sequence of *S. typhimurium* (GenBank AE008863), named as pVAX-asd. The porcine inhibin α (1–32) (86% homology with rat) and HBsAg-S recombinant gene fragment obtained from the pCIS plasmid (Invitrogen, Carlsbad, USA) was inserted into the NdeI and HindIII sites of the expression vector pVAX-asd, named as pVAX-asd-IS, and then transfected the reconstructed vector pVAX-asd-IS into attenuated *asd^-^* /*crp^-^ S. choleraesuis* C500 strain (C500/pVAX-asd-IS) and stroe at – 80°C.

### Vaccine identification and large-scale preparation

The C500 and C500/pVAX-asd-IS were recovered in ice, and cultured at 37°C overnight respectively. A single selected colony was inoculated in Luria–Bertani (LB) with antibiotics and cultured at 37°C for 1 h. The plasmids of C500/pVAX-asd-IS and C500 were extracted by Endotoxin-free plasmid extraction Kit (CWBIO, China), and sequenced by Shenzhen BGI Technology Co. LTD. Meanwhile, the plasmids were digested with *EcoRI* and *HindIII* (Ferments, China) for 1.5-2 hours, target fragment was detected using 1.5% agarose gel.

C500 and C500/pVAX-asd-IS were inoculated in LB using large bottles at 37°C for large-scale preparation of DNA vaccines, respectively. After 10h, the number of C500 and C500/pVAX-asd-IS in the bacterial solution was assessed using plate colony count method.

### Animals and vaccination

Forty healthy male Sprague–Dawley (SD) rats (6-weeks old, 267.5±12 g) were purchased from Hunan Silaikejingda Laboratory Animal Co. LTD., and randomly divided into four groups (n=10 per group), and respectively immunized with 1×10^8^, 1×10^9^, and 1×10^10^ CFU of the recombinant inhibin vaccine (group C500/pVAX-asd-IS-L, C500/pVAX-asd-IS-M, and C500/pVAX-asd-IS-H) or 1×10^10^CFU C500. The rats were administered orally and immunized thrice (same to primary immunization) with an interval of 14 d.

### Sample collection and measurements at decapitation

Blood samples were collected from the caudal vein on days 0, 7, 14, 21, 28, 35, and 42 after primary immunization, and centrifuged at 3000 rpm for 15 min at 4°C, then the serum was separated and stored at −80°C for further analysis.

After 42 days from primary immunization, all rats were anesthetized with phenobarbital sodium and then decapitated. Both testes were removed; epididymides were dissected and cut into pieces in 2 mL of 0.9% saline, filtered, the filtrate was used to determine sperm count and deformity rate as described previously (Nirmal et al., 2017). Analytical balances and vernier calipers were used to determine pairs of testicular weight, length and width. Testis volume was calculated using the formula: v = (4π (width/2)^2^(length/2))/4 and recorded as an average of both testes. Then, one testis was frozen in liquid nitrogen and stored at −80°C immediately for gene expression analysis, while the other was fixed in Bouin’s solution for histological analysis.

### Detection of antibodies against INH

Specific IgG antibodies of serum were determined using an indirect ELISA with synthetic INHα (1-32) antigens (Sigma, USA) as standard antigens on days 0, 7, 14, 21, 28, 35, and 42 after primary immunization according to previously described (Dan et al., 2016b). ELISA results were analyzed in terms of P/N ratios, P/N ratios higher than 2.0 and OD values higher than 0.2 were considered positive responses.

### Hormone determination

Serum concentrations of testosterone, inhibin B, LH and FSH were measured using rat-specific ELISA Kits (R&D Systems, USA) according to the manufacturer’s instructions. OD values were measured at 450 nm by a microplate reader. All samples were measured in thrice.

### Histological analysis of testes

The testes fixed in Bouin’s solution were dehydrated in a graded ethanol series from water through 70% to 100% ethanol in subsequent steps and embedded in paraffin. The samples were cut into 5 µm thick sections and stained with hematoxylin and eosin (HE). The slides were observed using a light microscope (Motic, China).

### Quantitative analysis of mRNA expression

Total RNA was isolated from testis using Column Animal RNAOUT kit (TIANDZ, China), according to the manufacturer’s instructions. The concentration of RNA was detected using Nanodrop-2000 spectrophotometer and the RNA integrity was evaluated by Agilent Bioanalyzer 2100. Total RNA (1μg) isolated from testis was used for cDNA synthesis through PrimeScript RT reagent Kit with gDNA Erase (TAKARA, Japan). Quantitative real-time PCR was performed using a 20 μL reaction volume containing 10 μL of SYBR Premix Ex Tap, 0.4 μL of forward and reverse primers, 0.4 μL of ROX Reference Dye (50X), 2 μL of cDNA and 7.2 mL RNase and DNase- free water. The reaction was run on a StepOne™ Real-Time PCR System (ABI, USA) set at 94°C for 30 s, 40 cycles of 94°C for 10 s, 60°C for 30 s and 72°C for 10 s. House-keeping gene β*-actin* was used as an internal control and the relative expression of the genes was calculated using the 2^-ΔΔCT^ method. A significant difference was statistically evaluated with Student’s t- test using SPSS 20.0. The primers used are listed in Table 1.

**Table 1:** Primer sequences of the three genes(INHβ-B, VIMENTIN and Smad4).

**Gene name**	**Primer sequences (5'→3')**	**Product length /bp**
β-actin	F:GGCTGTATTCCCCTCCATCG; R:CCAGTTGGTAACAATGCCATGT	154
INHβ-B	F:CGCGTCTCCGAGATCATACG; R:CACCACATTCCACCTGTCTCC	180
VIM	F:CGACGCCATCAACACCG; R:GCAACTCCCTCATCTCCTCC	206
SMAD4	F:ATCTATGCCCGTCTGTGGAG; R:AAGTTGGTGGTACTGGTGGC	362

### Statistical analysis

Differences in P/N value, growth performance, and serum hormone concentrations were analyzed by performing two-way analysis of variance (ANOVA) using the general linear models (GLM) procedure in the SPSS Statistics 24.0 software (SPSS Software, Inc., Chicago, IL). T esticular weight and volume, epididymal sperm count and deformity rate, and t esticular mRNA expressions of rat were analyzed using one-way ANOVA test followed by Dunnett’s multiple comparison test and significant difference was inferred for *p* < 0.05 and extremely significant difference *p* < 0.01. All data were presented as means ± SEM.

## Results

### Identification of vaccines

**Figure 1:**
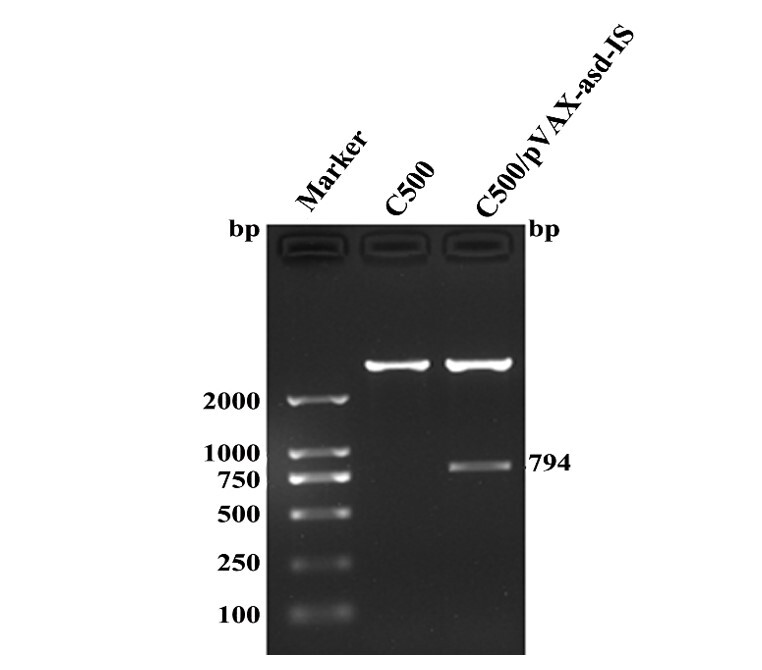
Identification of C500/pVAX-asd-IS and C500. Marker: DNA molecular weight markers (DL-2000); C500: C500 plasmids; C500/pVAX-asd-IS: the target fragment of C500/pVAX-asd-IS plasmids (794bp).

To verify the integrity of the DNA vaccines (C500/pVAX-asd-IS and C500), the plasmids were extracted and digested with *EcoRI* and *HindIII* endonucleases, and the target fragment of C500/pVAX-asd-IS plasmids (794bp) was displayed on 1.5% agarose gel, and the plasmids of C500 were not appeared (Fig.1). Meanwhile, sequence analysis showed that the C500/pVAX-asd-IS plasmids were intact.

### Detection of antibody in serum

**Figure 2:**
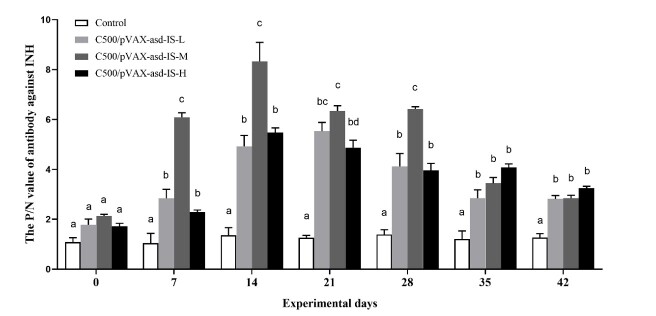
The P/N value of antibody against INH after primary immunization. Data are presented as the mean ± SEM (n = 10), Means for given bars, not sharing the same superscripts (a–d), are statistically significantly different, *p* < 0.05.

The rats immunized with C500/pVAX-asd-IS evidently induced antibodies against INH at 7 d after primary immunization. However, the anti-INH antibodies were not detectable in control group. Furthermore, the P/N values increased after vaccination and differed at 7 d (*p*<0.05). Following the booster vaccination, the P/N values sharply increased at 14 d (*p*<0.05). The results indicated that immunization of INH vaccines induced the immune response and production of anti-INH antibodies in rat. (Fig. 2)

### Growth performance

**Figure 3:**
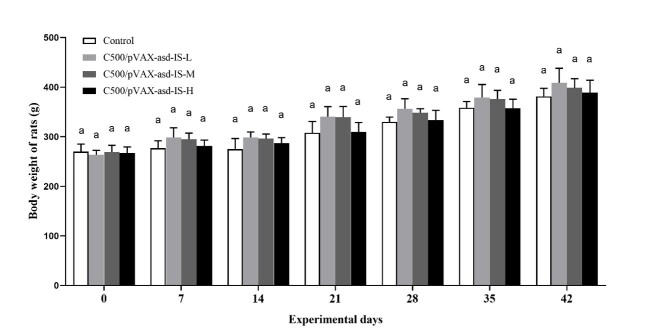
Effect of INH gene vaccine immunization on body weight of rats. Data are presented as the mean ± SEM (n = 10), Means for given bars, sharing the same superscripts (a), are not statistically significantly different, *p* > 0.05.

For the period between days 0 and 42, the body weight in C500/pVAX-asd-IS groups were greater than that of control group (*p* >0.05),and there was no significant difference in body weight among C500/pVAX-asd-IS groups (*p* > 0.05). (Fig. 3)

### Testicular weight and volume

**Figure 4:**
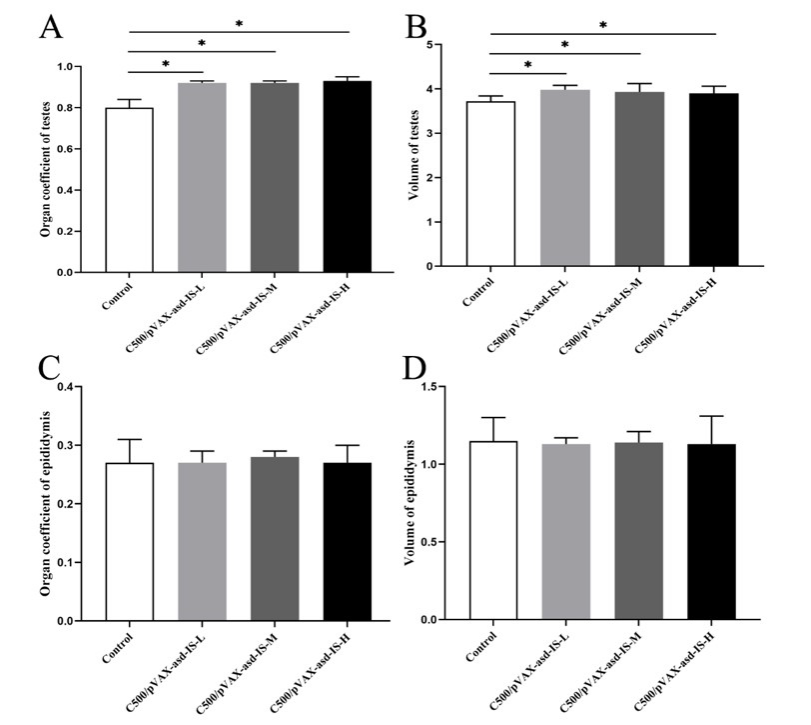
Effects of INH immunization on testicular organ coefficients (A), testicular volume (B), epididymal organ coefficients (C), and epididymal volume (D) of rats. Data are presented as the mean ± SEM (n = 10), * indicate significant difference *p* <0.05 among groups.

Compared to control group, immunization against INH promoted testis development, the weight and volume of testes in C500/pVAX-asd-IS groups were increased (*p* < 0.05) at decapitation, respectively. While epididymis, the weight and volume in C500/pVAX-asd-IS groups were not significantly affected by immunization (*p* > 0.05). (Fig. 4)

### Morphological observation of testes

**Figure 5:**
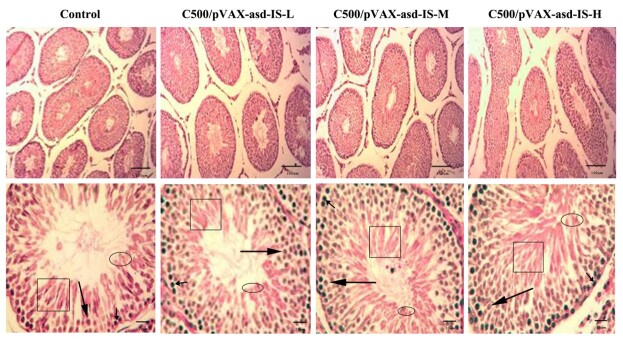
Morphological observation of testes. Cells in spermatogensis are shown as spermatogonia (short arrows), primary spermatocyte (long arrows), spermatid (ellipse), and spermatozoa (square). The figure above – magnification ×100, scale bar = 100 µm; the figure below – magnification ×400, scale bar = 20 µm. Testicular tissues were stained with H and E.

HE staining did not reveal significant difference to the seminiferous tubules in the C500/pVAX-asd-IS-L group. In C500/pVAX-asd-IS-M and C500/pVAX-asd-IS-H groups, we noticed the basement membrane of seminiferous tubules was thickened, the space between tubules was narrowed, the lumen of seminiferous tubules became larger, the number of spermatogenic cells in the lumen increased, the sperm count increased, and the cytoplasmic remnants of sperm allergy increased compared with the control group. (Fig. 5)

### Sperm count and deformity rate in epididymis of rat

**Figure 6:**
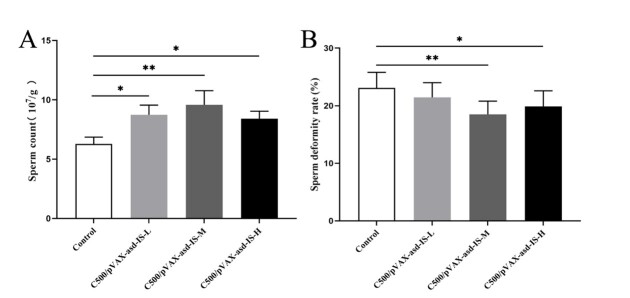
Effects of INH immunization on sperm count(A) and deformity rate(B) in epididymis of rat. Results were compared with the control group and data are presented as the mean ± SEM (n = 10), * and ** indicate significant difference *p* <0.05 and *p* <0.01 respectively among groups.

The count of sperm in epididymis was calculated using a hemocytometer, and the sperm count was greater in C500/pVAX-asd-IS groups compared with that in control group (*p* < 0.05), especially, the difference between C500/pVAX-asd-IS-M and the control group was extremely significant (*p* < 0.01). (Fig. 6A)

The sperm deformity rate of C500/pVAX-asd-IS was less than that of the control group, and the sperm deformity rate of C500/pVAX-asd-IS-M was extremely significantly less than that of the control group (*p* < 0.01), the sperm deformity rate of C500/pVAX-asd-IS-H was significantly less than that of the control group (*p* < 0.05).(Fig. 6B)

### Serum hormone concentrations

**Figure 7:**
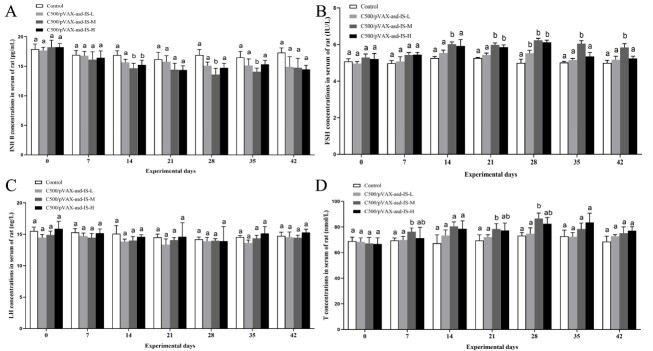
Effects of INH immunization on serum INH B (A), FSH (B), LH (C), and T (D) concentrations of rats. Data are presented as the mean ± SEM (n = 10), Means for given bars, not sharing the same superscripts (a–b), are statistically significantly different, p < 0.05.

On day 14 after the first immunization, serum INH B concentration in C500/pVAX-asd-IS-M and C500/pVAX-asd-IS-H group declined markedly compared with control group (p < 0.05); and a significantly decrease was observed for INH B amounts on day 28, 35 after the first immunization (p < 0.05) (Fig. 7A). Compared with the control group, the serum FSH concentration in C500/pVAX-asd-IS-M group presented an increasing tendency on day 14, 21, 28, 35, and 42 after the first immunization; levels of FSH also improved in C500/pVAX-asd-IS-H group compared with the control group (p < 0.01) on day 21 and 28 after primary immunization (Fig. 7B). For the LH concentration in serum, the results revealed that there was no significant difference among groups (p > 0.05) (Fig. 7C). Serum T concentrations in the C500/pVAX-asd-IS-M group were significantly greater than that of the control group (p < 0.05) on day on day 7, 21, and 28 after the first immunization (Fig. 7D).

### mRNA expressions

**Figure 8:**
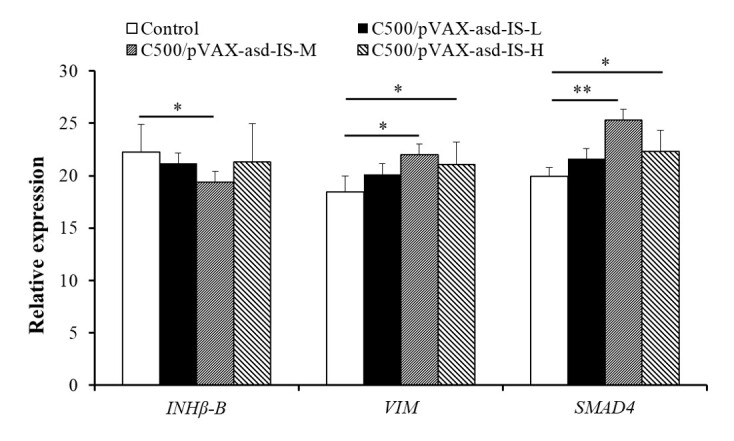
Relative expression of spermatogenesis related genes in testes of rats. Results were compared with the control group and data are presented as the mean ± SEM (n = 10), * and ** indicate significant difference *p* <0.05 and *p* <0.01 respectively among groups.

The mRNA expressions for spermatogenesis related genes in in testes of rats are shown in Fig. 8. Compared to control group, *VIM* and *SMAD4* expressions were increased in C500/pVAX-asd-IS-M and C500/pVAX-asd-IS-H groups (*p* < 0.05 or *p* < 0.01). The mRNA amount of *INHβ-B* in C500/pVAX-asd-IS-M group was greater than control group (*p* < 0.05).

## Discussion

Over the past decade, the use of recombinant attenuated Salmonella vaccine strains for heterologous antigen delivery has increased considerably. In the mucosal immunization routes, the oral route of antigen delivery is the most common and frequently explored, which stimulates both systemic and mucosal immune responses (Zhao et al., 2008). The oral DNA vaccine against Giardia using attenuated Salmonella and carrying CWP2-DNA possesses the satisfied immunogenicity and effectively stimulates the body to produce systemic immunity (IgG) and local mucosal immunity (SIgA) (Zhang et al., 2015). In this study, all rats immunized orally with C500 or C500/pVAX-asd-IS survived, and no signs of disease were observed in the immunized mice throughout the experimental period. In our results, oral inoculation of INH DNA vaccines induced the immune response and production of anti-INH antibodies in rat. The attenuated Salmonella vaccine was administered orally into the intestines and engulfed by phagocytes in Peyer's lymph node in animals, the plasmids were released and transported to the cytosol, where they are integrated into the nucleus, and finally express INH in host cells (Loessner & Weiss, 2004).

INH exhibits a vital role on the hypothalamus-pituitary gonadal (HPG) axis (Rehman et al., 2021). Immunization against INH has been used in mammalian species to improve reproductive efficiency and spermatogenesis (Akhtar et al., 2021). Numbers of sperm started increasing after INH immunization in merino rams (Voge & Wheaton, 2007). Active immunization against INH for bulls, the sperm density was increased, and the number of sperm was increased after the booster immunization (Schanbacher, 1991). Our results showed that INH gene vaccine immunization increased the number of sperm and decreased the sperm deformity rate in rats. The spermatogenic function of testis was positively correlated with its volume, and the spermatogenic dysfunction increased with the reduction of testicular volume (Todorić et al., 2014). In this study, the volume and weight of testis were significantly increased after oral administration of INH gene vaccine. In terms of morphology, the number of spermatogenic cells and the number of layers were relatively increased, and the exfoliated cells in the lumen of tubules were also significantly increased. The number of spermatogenic cells can be explained by the accentuated role of INH (Van Dissel-Emiliani et al., 1989). The presence of ascending numbers of apoptotic cells in C500/pVAX-asd-IS groups enhances importance for normal spermatogenesis.

Apoptosis is a normal physiological phenomenon during testis steroidogenesis (Banerjee & Chaturvedi, 2017). On hypothalamus pituitary gonadal axis, LH and FSH are under positive control of hypothalamic GnRH and gonadal steroid and INH, that exert positive and negative feedback effects on gonadotropins either directly or via hypothalamic GnRH (Themmen & Huhtaniemi, 2000). Previous studies have shown that INH immunization increases FSH concentrations and stimulates follicular development by neutralizing endogenous INH (Wang et al., 2012). Similarly, our study indicated that INH immunization has improved the FSH and T levels. INH immunization produces specific antibodies to neutralize endogenous INH, which promotes the FSH secretion and inhibits LH secretion in a feedback manner (Mitchell et al., 2011). Furthermore, Serum INHB concentrations were decreased in C500/pVAX-asd-IS groups after the booster immunization. In agreement with our finding, it was reported that serum INH B concentrations were also significantly decreased in rats after INH antagonist administration (Akhtar et al., 2019). In this process, the inhibitory effect of INHB on FSH secretion was stronger than the positive feedback effect (Johansen et al., 2014), and the decreased secretion of INHB may regulate and promote the synergistic effect between sertoli cells and germ cells through paracrine, and promoting spermatogenesis. Testicular leydig cells secrete a large amount of testosterone, which promotes and maintains spermatogenesis and testes development (Han et al., 2016).

In the previous study, VIM was identified as a biomarker which involved in seminiferous tubules structure and spermatogenesis (Pecile et al., 2021). VIM regulates various cellular functions through different pathways and plays an important role in the environment of spermatogenesis in male animals (Albert et al., 2012). SMAD4 is absolutely required for FSH synthesis in mice (Fortin et al., 2014). The *SMAD4* mRNA was found to be highly expressed in sertoli cells or stromal cells of adult mice, and it is demonstrated that regulating SMAD mRNA and protein expression is a feature of mouse testis development and spermatogenesis (Itman & Loveland, 2008). Testis is underdeveloped during embryonic period in *SMAD4* knockout mice, which indicates that this gene plays an important role in testicular development (Archambeault & Yao, 2014).The severely arrested spermatogenesis was accompanied by the significantly decreased mRNA expressions of INHβB in testes (Han et al., 2016), the decrease of INHβB mRNA expressions may lead to the function lesions of testis (Han et al., 2015). In this study, the increased sperm count in C500/pVAX-asd-IS groups, the decreased malformation rate in C500/pVAX-asd-IS-M and C500/pVAX-asd-IS-H groups, the down-regulation of *InHβB* mRNA in C500/pVAX-asd-IS-M, and the up-regulation of *VIM* and *SMAD4* in C500/pVAX-asd-IS-M and C500/pVAX-asd-IS-H groups indicating that INH immunization may have a positive effect on spermatogenesis in male rats.

## Conclusion

This study characterized that neutralization of endogenous INH through oral vaccination with INH DNA delivered by C500 strain successfully elicited a humoral immune response. Oral vaccination with INH DNA vaccine increased testicular weight, volume, spermatogenic cell count in the lumen, sperm cytoplasmic remnants. Oral vaccination with INH DNA vaccine reduced INHB concentrations, enhanced T and FSH concentrations in serum. Furthermore, Oral vaccination with INH DNA changed the *INHβ-B*, *VIM*, and *SMAD4* mRNA expressions. Therefore, INH immunization may have a positive effect on spermatogenesis and reproductive efficiency in male rats.
